# Systemic Sclerosis, Malnutrition, and Small Bowel Obstruction: Why Clinicians Should Consider Early Total Parenteral Nutrition in Systemic Sclerosis With Severe Gastrointestinal Involvement

**DOI:** 10.7759/cureus.27638

**Published:** 2022-08-03

**Authors:** Ben Massat, James McCarthy

**Affiliations:** 1 Internal Medicine, Medical College of Wisconsin, Wauwatosa, USA

**Keywords:** intestinal dysmotility, small intestinal bacterial overgrowth, acute colonic pseudo-obstruction, s:malnutrition, small-bowel obstruction, diffuse systemic sclerosis

## Abstract

Systemic sclerosis can cause vascular endothelial damage and fibrosis involving nearly all aspects of the gastrointestinal tract. This can lead to esophagitis, gastroparesis, small bowel dysmotility, small intestinal bacterial overgrowth, chronic intestinal pseudo-obstruction, and malnutrition among other complications. We present a case of a 62-year-old woman with a history of diffuse cutaneous systemic sclerosis who developed significant gastrointestinal involvement, leading to multiple mechanical small bowel obstructions and severe malnutrition. Several previously published case reports have documented pseudo-obstruction in systemic sclerosis, rather than mechanical small bowel obstruction. This case underscores the importance of evaluating for mechanical small bowel obstruction in patients with systemic sclerosis prior to initiating treatment for pseudo-obstruction. It also highlights that in patients with nutritional deficiencies secondary to systemic sclerosis with gastrointestinal involvement, early initiation of total parenteral nutrition should be strongly considered.

## Introduction

Systemic sclerosis, an autoimmune disorder characterized by endothelial damage and fibroblast activation leading to excessive collagen deposition in the skin and internal organs [1], can lead to devastating gastrointestinal complications. Patients can develop vascular endothelial damage and fibrosis involving nearly every aspect of the gastrointestinal tract including the oral cavity, esophagus, stomach, small bowel, colon, and liver, which often leads to malnutrition [2-3]. We present a case of systemic sclerosis complicated by severe malnutrition and multiple small bowel obstructions.

This article was previously presented as a poster at the American College of Physicians Wisconsin Chapter Scientific Meeting on September 10, 2021.

## Case presentation

A 62-year-old woman with a history of diffuse cutaneous systemic sclerosis presented with a day's duration of severe lower abdominal pain, an inability to tolerate oral intake, and no bowel movements during this time. Her systemic sclerosis had been diagnosed approximately one and a half years prior to the presentation when she had developed unintentional weight loss, Raynaud’s phenomenon, dyspnea on exertion, dysphagia, a papular rash of the hands and feet, and arthralgias. Antinuclear antibody was positive at a titer of 1:2560 in a nucleolar pattern and a forearm biopsy revealed dermal sclerosis with mild lymphoplasmacytic inflammation consistent with systemic sclerosis. Additional significant manifestations of her systemic sclerosis noted prior to her hospital presentation included skin thickening, mild lung parenchymal scarring, and esophageal dysmotility. Her most recently documented modified Rodnan skin score (mRSS) was 25. Of note, cardiac involvement, pulmonary hypertension, digital ulcerations, and history of any renal hypertensive crisis were absent. She had been receiving treatment with hydroxychloroquine 200 mg daily, mycophenolate sodium 720 mg twice daily, and prednisone 5 mg daily.

Physical exam on presentation was significant for abdominal distention with epigastric tenderness, and CT of the abdomen and pelvis showed a high-grade distal small bowel obstruction with a transition point in the terminal ileum. She was admitted and treated conservatively with nasogastric tube decompression, fluid resuscitation, and pain control with the resolution of the obstruction. Due to persistent nausea and vomiting while advancing her diet, she underwent esophagogastroduodenoscopy (EGD), which showed erythematous mucosa at the antrum, gastric body, and gastroesophageal junction as well as signs of slow gastric motility suspicious for gastroparesis. These findings likely contributed to her nausea and vomiting, and tolerance to oral intake improved with scheduled metoclopramide. After discharge, esophageal biopsies showed evidence of Candida esophagitis and she was started on a three-week course of fluconazole.

Prior to finishing her course of fluconazole, the patient again presented with worsening weakness and signs of malnutrition including a 10-pound weight loss over a one-month period, cachexia, and muscle wasting. She reported significant difficulties with activities of daily living for one week as well as continued weight loss, totaling 50 pounds over the past year. She endorsed dysphagia to solid foods but denied any with liquids. Initial laboratory evaluation revealed a magnesium level of 1.3 mg/dL and an albumin level of 3.2 g/dL but was otherwise without any significant electrolyte abnormality or other metabolic derangements. A video swallow study showed oropharyngeal and esophageal dysphagia with no signs of aspiration. EGD revealed retained food in the stomach and duodenal aspirate culture showed a value of >1,000,000 CFU/mL, consistent with small intestinal bacterial overgrowth (SIBO). The patient declined to begin enteral nutrition despite recommendations, and she showed modest improvement in oral intake. She was discharged home with a prescription for rifaximin 550 mg three times daily for two weeks.

**Figure 1:**
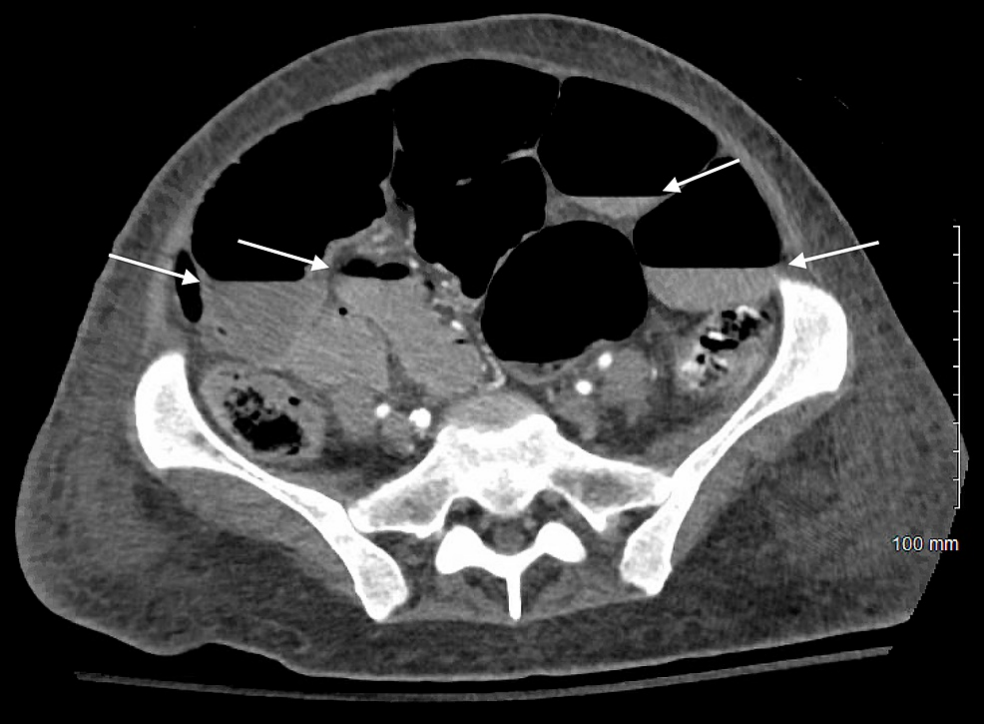
CT image of air-fluid levels from the patient's second high-grade distal small bowel obstruction (arrows) CT: computed tomography

Two days after discharge, the patient was again admitted with continued diet intolerance and malnutrition. Nasogastric feedings were initiated; however, she experienced severe nausea and vomiting as well as significant gastric residual volumes. Repeat EGD showed retained fluid in the esophagus, esophageal ulcerations, and retained food in the duodenum suggesting small bowel dysmotility, and a nasojejunal tube was placed. Once nasojejunal feedings were started, the patient developed significant abdominal distention and a CT of the abdomen and pelvis showed a high-grade small bowel obstruction with a transition point in the distal ileum (Figure 1).

Per surgery recommendations, the patient was treated with bowel rest and nasogastric decompression. Total parenteral nutrition with 20% lipid emulsion was started for nutritional support. Two days later, she developed pulmonary edema and then went into pulseless electrical activity arrest. She was transitioned to a comfort-centered plan of care and died shortly thereafter.

## Discussion

Our patient had many gastrointestinal manifestations of systemic sclerosis including Candida esophagitis, esophageal dysmotility, gastroparesis, small bowel dysmotility, and SIBO. She also repeatedly developed small bowel obstruction, a unique aspect of her presentation given that systemic sclerosis is more typically associated with pseudo-obstruction [2-7]. In addition to multiple reports of pseudo-obstruction, a review of previously published cases showed rare instances of volvulus leading to obstruction, with cases involving the small bowel and colon [7-9]. Our patient’s case is unique in that she suffered from two separate instances of mechanical obstruction without volvulus, both in the terminal ileum. Her significant small bowel dysmotility and history of abdominal hysterectomy both likely contributed to the development of these small bowel obstructions, underscoring the importance of uncovering risk factors and evaluating for mechanical small bowel obstruction with abdominal CT in systemic sclerosis patients prior to treatment with an acetylcholinesterase inhibitor for presumed pseudo-obstruction.

In addition to increasing her risk of small bowel obstruction, our patient’s gastrointestinal manifestations of systemic sclerosis significantly contributed to her malnutrition. Her esophageal dysmotility, gastroparesis, and SIBO made tolerating any form of oral or enteral feeds impossible. Considering our patient’s poor outcome, we argue that total parenteral nutrition should be considered as an early intervention for patients with nutritional deficiencies secondary to systemic sclerosis with gastrointestinal involvement, especially in those with additional risk factors for small bowel obstruction. This may help increase awareness and avoid the morbidity and mortality associated with developing both mechanical small bowel obstruction and pseudo-obstruction in these patients.

## Conclusions

This case underscores the importance of uncovering additional risk factors of small bowel obstruction in patients with systemic sclerosis as well as evaluating with abdominal CT prior to treating for pseudo-obstruction. It demonstrates that despite the propensity for systemic sclerosis to lead to pseudo-obstruction, patients can still develop mechanical small bowel obstruction for which neostigmine would be contraindicated. While this patient had extensive complications related to her systemic sclerosis, her clinical decline coincided with her nutritional deterioration and lack of physiologic reserve. We argue that in patients with nutritional deficiencies secondary to systemic sclerosis with gastrointestinal involvement, total parenteral nutrition should be considered sooner, especially during periods of acute illness. We suspect that systemic sclerosis patients with gastrointestinal complications may greatly benefit from early total parenteral nutrition given the endothelial damage and fibrosis to nearly every aspect of the gastrointestinal tract, which makes it difficult to maintain adequate nutritional support with oral or enteral feeds alone.
